# Src: coordinating metabolism in cancer

**DOI:** 10.1038/s41388-022-02487-4

**Published:** 2022-10-10

**Authors:** Sara G. Pelaz, Arantxa Tabernero

**Affiliations:** Instituto de Neurociencias de Castilla y León (INCYL), Departamento de Bioquímica y Biología Molecular, Universidad de Salamanca, Instituto de Investigación Biomédica de Salamanca (IBSAL), Calle Pintor Fernando Gallego 1, Salamanca, 37007 Spain

**Keywords:** Cancer metabolism, Oncogenes

## Abstract

Metabolism must be tightly regulated to fulfil the dynamic requirements of cancer cells during proliferation, migration, stemness and differentiation. Src is a node of several signals involved in many of these biological processes, and it is also an important regulator of cell metabolism. Glucose uptake, glycolysis, the pentose-phosphate pathway and oxidative phosphorylation are among the metabolic pathways that can be regulated by Src. Therefore, this oncoprotein is in an excellent position to coordinate and finely tune cell metabolism to fuel the different cancer cell activities. Here, we provide an up-to-date summary of recent progress made in determining the role of Src in glucose metabolism as well as the link of this role with cancer cell metabolic plasticity and tumour progression. We also discuss the opportunities and challenges facing this field.

## Graphical abstract

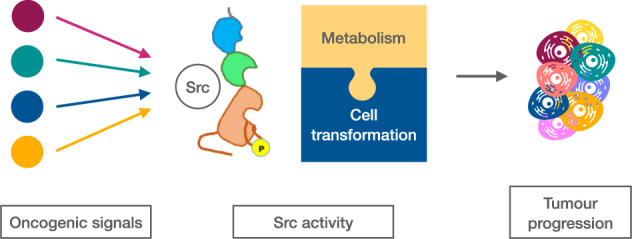

## Introduction

The hallmarks of cancer—shared commonalities that unite all types of cancer cells at the level of cellular phenotype—have been recently updated [1]. They comprise the acquired capabilities for sustaining proliferative signalling, evading growth suppressors, resisting cell death, enabling replicative immortality, inducing/accessing vasculature, activating invasion and metastasis, avoiding immune destruction and deregulating cellular metabolism [1].

Src, one of the best studied oncoproteins, has been shown to regulate these hallmarks that ultimately control the behaviour of transformed cells and contribute to tumour progression and metastasis [2]. Excellent reviews with comprehensive information about Src substrates and the broad spectrum of cellular events regulated by this kinase were published during those years in which an exponential growth of the research in the field took place [3, 4]. However, the effects of Src on metabolism and their relevance on cell transformation was not yet uncovered at the time. Importantly, “deregulating cellular metabolism” has been recently included as a core hallmark of cancer [1]. Because of the extensive number of studies showing a role of Src in metabolism, in this review we will summarise the effects of Src on glucose metabolism, and discuss their contribution to Src oncogenic activity and the related therapeutic opportunities. Before addressing this topic, we will introduce Src family kinases, their structural properties, as well as the regulation of their activity and oncogenic properties. We will focus on Src, the best studied and prototypical member of the family and we will refer to other Src family members when necessary.

## Src structure and regulation

The seminal discovery that the transforming element of the Rous sarcoma virus (*v*-*src*) in chickens was a transduced form of the cellular gene *c-src* gave rise to the identification of the first oncogene and proto-oncogene [5]. c-Src (herein termed Src) is the founding member of the Src family of non-receptor protein tyrosine kinases (SFKs), which are key regulators of signal transduction implicated in fundamental cellular processes, many of them related to human cancers [6]. SFKs include Src, Fyn, Yes, Lck, Hck, Blk, Lyn, Fgr and Frk. Src, Fyn and Yes are ubiquitously expressed, while Lyn, Hck, Fgr, Blk and Lck are predominantly and differentially expressed in the various cell types of the haematopoietic lineage. Although some unique functions have been reported for some SFK members, extensive functional redundancy among SFKs exits in different cell types [7, 8].

**Fig. 1:**
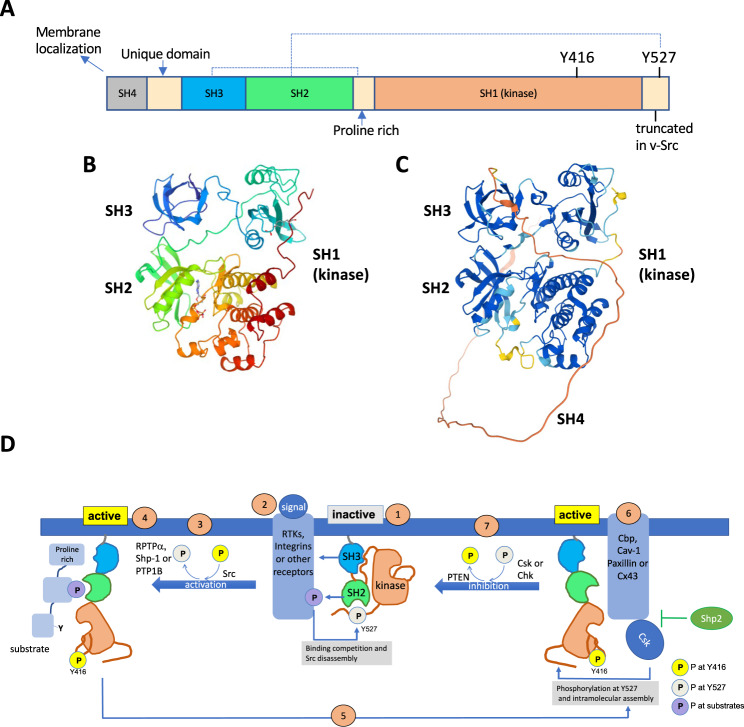
Src structure and regulation. **A** The domain structure of Src. Dashed lines depict intramolecular contacts established in the inactive conformation. **B** Three-dimensional structure of Src [145] (https://www.rcsb.org/structure/1FMK). **C** Src structure prediction with AlphaFold [144]. The comparison between these structures indicates the accuracy of AlphaFold prediction, which might be very useful for future studies of Src interactome, structure-function relationship and design of specific inhibitors. Note that B does not include the disordered SH4 region, which is shown in red in **C**. **D** Regulation of Src activity. (1) Src in the inactive, “closed” or assembled conformation with intramolecular contacts of the SH2 domain with phosphorylated Tyr527 and the SH3 domain with the proline rich region. (2) Src is activated in response to diverse signals; activated receptors compete for binding to Src SH2 or SH3 domains and disrupt Src intramolecular interactions. (3) Src is activated by autophosphorylation at Tyr416 and dephosphorylation at Tyr527, catalysed by the receptor protein tyrosine phosphatase α (PTPRA), the nonreceptor tyrosine phosphatase SHP-1 (PTPN6) or PTP1B. (4) Src in the active “open” or disassembled conformation with phosphorylated Tyr416. Src interacts with the SH3 and SH2 domain to the proline rich region and phosphotyrosine motif within the substrate for tyrosine phosphorylation. (5) Inhibition of Src requires the binding of active Src to CSK recruiting proteins (Cbp, Cav-1, Paxillin or Cx43). SHP-2 inhibits CSK recruitment. (6) CSK phosphorylates Src at Tyr527 promoting its intramolecular interaction with the SH2 domain. (7) Phosphatases, such as PTEN, remove phosphate at Tyr416 causing full Src inactivation (1).

SFKs share a conserved domain structure (Fig. 1A–C) composed of the SH4 region, which contains the lipidation site (mainly myristylation site) for membrane localisation; a unique domain characteristic of each individual kinase; the SH3 domain, which binds proline-rich sequences; the SH2 domain, which binds phosphotyrosine-containing sequences; the SH1 domain, which is the catalytic kinase domain and contains the substrate- and the ATP- binding site, as well as the autophosphorylation site (Tyr416 in chicken Src, the most used terminology because of its discovery and Tyr419 in human Src); and a short C-terminal tail, which contains the negative-regulatory tyrosine residue (Tyr527 in chicken Src and Tyr530 in human Src). All family members show extensive sequence homology in the SH1, SH2, and SH3 domains and in the SH4 region, but diverge in the unique domain (reviewed in [9]).

SFKs have multiple regulatory mechanisms, which converge on tyrosine phosphorylation at two sites – residues Tyr416 and Tyr527 for Src and nearby for other SFK members—with opposing effects. Because most of the regulatory mechanisms are shared by SFKs, we will refer to Src as the prototypical member of this family. Phosphorylation of Tyr416, located within the activation loop of the kinase domain (SH1), activates the enzyme while phosphorylation of Tyr527, located within the C-terminal tail, inhibits enzymatic activity (Fig. 1D). In resting cells, Src is maintained in an inactive conformation, in which intramolecular contacts of the SH2 domain with phosphorylated Tyr527 and that of the SH3 domain with the proline rich region (Fig. 1A) cause an assembled or closed state (Fig. 1D). The oncogenic v-Src protein lacks the inhibitory Tyr527 site and that is why it is constitutively active and highly transforming. Thus, although v-Src and c-Src are proposed to have the same substrates, the lack of regulation in v-Src causes the permanent activation and the subsequent neoplastic transformation, while c-Src is not transforming unless mutated, overexpressed or overactivated.

Src is activated in response to a great diversity of extracellular signals via integrins [10], G-protein-linked receptors [11], steroid receptors [12], and receptor tyrosine kinases (RTKs) [13], such as platelet-derived growth factor receptor (PDGFR), epidermal growth factor receptor (EGF-R) family, fibroblast growth factor receptor (FGF-R), insulin-like growth factor-1 receptor (IGF-1R), c-Met, colony-stimulating factor-1 receptor (CSF-1R) and stem cell factor receptor (SCF-R), among others [13]. Although the participation of some intermediates, such as Ral-GTPase, has been reported [14], the aforementioned receptors compete for binding to Src SH2 or SH3 domains and disrupt the intramolecular interactions, allowing for Src kinase activation. For instance, PDGFR binds to Src SH2 domain while ß3 subunit of integrins binds to Src SH3 domains (reviewed in [15]). The disassembly of intramolecular contacts allows autophosphorylation at Tyr416 [16] and dephosphorylation at Tyr527, which can be catalysed by the receptor protein tyrosine phosphatase α (PTPRA) [17], the nonreceptor tyrosine phosphatase SHP-1 (*PTPN6*) [18] or PTP1B [19]. Src adopts an open conformation in which the Src SH3 domain interacts with the proline rich region and the Src SH2 domain with the phosphotyrosine site, both at the target substrate. The Src kinase domain is then in the competent position for the phosphorylation of the specific tyrosine within the substrate (Fig. 1D).

The inhibition of Src requires the activity of the C-terminal Src kinase (CSK) [20] or CSK homolog kinase (Chk/MATK) [21], which phosphorylate Src at Tyr527. In addition, several phosphatases, such as phosphatase and tensin homolog (PTEN), have been shown to dephosphorylate Src at Tyr416 [22, 23], a step required to complete the inactivation and intramolecular assembly of Src (Fig. 1D). Whereas Src is a membrane-associated protein, CSK does not contain a membrane-binding motif and requires a membrane-anchor protein, such as the CSK binding protein or phosphoprotein associated with glycosphingolipid-enriched microdomains (Cbp/PAG). The interaction of CSK to Cbp/PAG is required for CSK to catalyse the phosphorylation of Src at Tyr527 causing Src inhibition. Interestingly, other membrane-associated proteins, such as caveolin-1 [24, 25], paxillin [26] and connexin43 [27] have the ability to recruit CSK and Src, favouring Src inhibition. Connexin43 can recruit CSK together with PTEN, which allows a cooperative and complete inhibition of Src [27]. This cooperative mechanism is also found in haematopoietic cells, where CSK interacts with the phosphatase encoded by *PTPN22*, which dephosphorylates Tyr394 of Lck and Tyr417 of Fyn (the equivalent to Tyr416 in Src) to inhibit Lck and Fyn activity concomitantly with CSK [28]. Furthermore, phosphorylation of Cbp/PAG facilitates the binding and activity of CSK. The protein-tyrosine phosphatase SHP-2 (PTPN-11) prevents Src inhibition by removing the phosphorylation of Cbp/PAG, thereby inhibiting CSK recruitment and access to Src [29]. On top of that, other mechanisms, such as the oxidation of key cysteine residues within the Src protein, can contribute to the regulation of Src activity [30, 31]. In brief, the regulation of Src activity is a complex process that involves a dynamic conformational transition in which the crosstalk of multiple signalling molecules takes part.

## The role of Src activity in cancer

### Src activity

The relevance of Src activity can be inferred by the presence of *src* genes across the whole range of metazoan evolution [3]. Src catalyses tyrosine phosphorylation at specific positions in a wide variety of proteins, regulating their activity. Among the proteins that have been found proposed to be Src substrates are those receptors mentioned previously that activate Src activity, as they can also be reciprocally activated by Src; transcription factors, such as Stat3 [32]; adaptor proteins, such as Shc [33], which leads to the subsequent activation of the Ras/Raf/Erk signalling cascade; other kinases, such as phosphoinositide 3-kinases (PI3Ks) [34], MAPK [34] or Akt [35]; channel proteins involved in cell communication, such as connexin43 [36] or pannexin1 [37]; cytoskeleton components such as FAK, p130 CAS, cortactin, paxillin or p190 Rho-GTPase-activating protein (GAP) (for a review, see [38]), and key metabolic enzymes that will be described in detail the following sections. As a consequence of these direct phosphorylations, extensive proteins and signalling pathways can be secondarily affected, including cyclin D1 or HIF-1ɑ [39]. Because Src functions as both effector and regulator of a plethora of receptors, this kinase facilitates the crosstalk between different signalling pathways. Src is, therefore, a node of communication in a complex network of interacting proteins [40], which can regulate many cellular events, including proliferation, differentiation, survival, migration, cytoskeletal organisation, adhesion, cell communication, stemness and metabolism. Despite the high diversity of Src effectors, it is important to keep in mind that Src activity can be used differently by individual extracellular stimuli, contributing to their ability to generate unique cellular responses in a context-dependent manner [14].

### Src in cancer

**Fig. 2:**
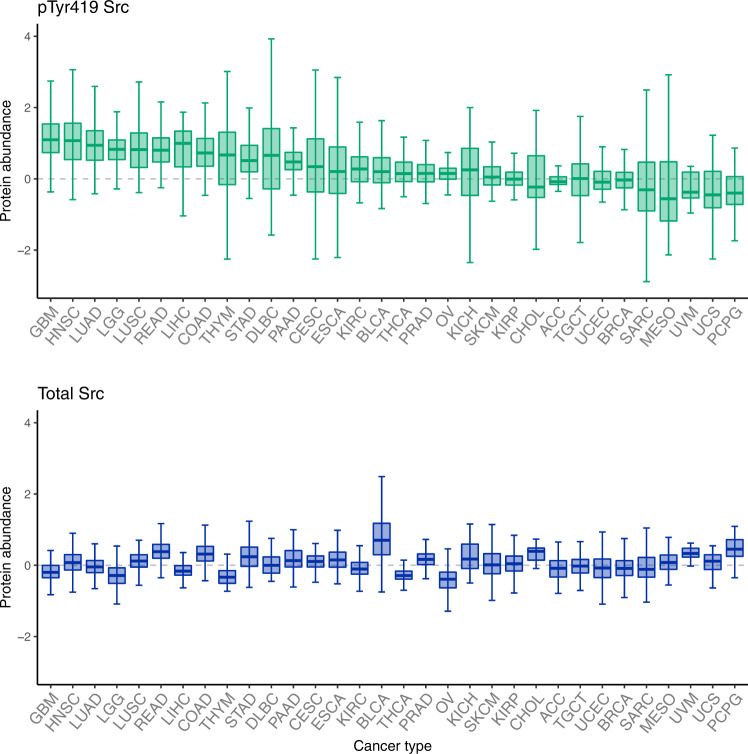
Reverse phase protein array (RPPA) abundance of active Src (phosphorylated at Tyr419) and total Src across different cancer types. Data obtained from The Cancer Proteome Atlas [146]. Cancer types are ranked by phosphorylated Tyr419 (upper panel) and the corresponding total Src abundance is shown in the bottom panel. Data were generated by the TCPA by first normalising protein values across samples, then normalising sample values across proteins, and finally combining data across multiple batches after a replicate-based normalisation (see https://tcpaportal.org/tcpa/faq.html and [146]). The plots were generated with R (v 4.1.2) [147]. The whiskers are the minimum and maximum value within 1.5 times the interquantile range under or over the 25th and 75th percentile respectively, the lower and upper hinges correspond to the first and third quartiles (the 25th and 75th percentiles), the bar corresponds to the median and the outliers are not shown. Adrenocortical carcinoma (ACC, *n* = 46), Bladder Urothelial Carcinoma (BLCA, *n* = 344), Breast invasive carcinoma (BRCA, *n* = 874), Cervical squamous cell carcinoma and endocervical adenocarcinoma (CESC, *n* = 171), Cholangiocarcinoma (CHOL, *n* = 30), Colon adenocarcinoma (COAD, *n* = 357), Lymphoid Neoplasm Diffuse Large B-cell Lymphoma (DLBC, *n* = 33), Esophageal carcinoma (ESCA, *n* = 126), Glioblastoma multiforme (GBM, *n* = 205), Head and Neck squamous cell carcinoma (HNSC, *n* = 346), Kidney Chromophobe (KICH, *n* = 63), Kidney renal clear cell carcinoma (KIRC, *n* = 445), Kidney renal papillary cell carcinoma (KIRP, *n* = 208), Brain Lower Grade Glioma (LGG, *n* = 427), Liver hepatocellular carcinoma (LIHC, *n* = 184), Lung adenocarcinoma (LUAD, *n* = 362), Lung squamous cell carcinoma (LUSC, *n* = 325), Mesothelioma (MESO, *n* = 61), Ovarian serous cystadenocarcinoma (OV, *n* = 411), Pancreatic adenocarcinoma (PAAD, *n* = 105), Pheochromocytoma and Paraganglioma (PCPG, *n* = 80), Prostate adenocarcinoma (PRAD, *n* = 351), Rectum adenocarcinoma (READ, *n* = 130), Sarcoma (SARC, *n* = 221), Skin Cutaneous Melanoma (SKCM, *n* = 353), Stomach adenocarcinoma (STAD, *n* = 392), Testicular Germ Cell Tumours (TGCT, *n* = 118), Thyroid carcinoma (THCA, *n* = 372), Thymoma (THYM, *n* = 90), Uterine Corpus Endometrial Carcinoma (UCEC, *n* = 404), Uterine Carcinosarcoma (UCS, *n* = 48), Uveal Melanoma (UVM, *n* = 12).

Since the seminal discovery of the transforming ability of v-Src, the role of Src in cancer has been extensively studied: Src is the SFK that is most often implicated in cancer. Indeed, although mutations in Src are a rare event, both overexpression and overactivation of Src have been observed in numerous cancer types, including those of the brain, mainly glioblastoma (GBM), as well as cancer of the liver, lung, colon, breast, bladder and pancreas, contributing to their malignancy grade (Fig. 2, reviewed in [41]). The increased Src activity found in cancer cells can be caused by multiple factors, including an enhanced expression of Src activators, frequently found in cancer, such as integrins [10], EGFR [14, 42], the constitutively active mutant form EGFRvIII [43], HER2 or ErbB2 [44] or other RTKs. Alternatively or concomitantly, downregulation of CSK [45], upregulation of SHP-2 or alterations in other Src regulatory molecules can contribute to the increased Src activity found in many cancers [46]. As described previously, Src integrates and regulates receptor signalling and directly transduces it to downstream effectors affecting many cellular events related to cell transformation, including metabolism, proliferation, differentiation, apoptosis, cell adhesion, migration, invasion, stemness and metastasis. Src may also play a prominent role in the tumour microenvironment, by inducing angiogenesis [43] or immune evasion [47]. Definitely, the study of Src activity and its target proteins will help to understand the biology of cancer, as well as its diagnosis and prognosis. For instance, the detection of site-specific phosphorylation levels of Src target proteins in peripheral circulating exosomes might be informative in cancer diagnosis and/or prognosis.

### Src participates in the phenotypic plasticity of cancer stem cells

Cancer stem cells (CSCs) or tumour-initiating cells are a subpopulation of undifferentiated tumour cells with distinct stem cell-like features, such as self-renewal and phenotypic plasticity, an emerging cancer hallmark [1]. CSCs are involved in metastasis, tumour recurrence and are highly resistant to conventional therapies [48]. Although multiple signalling pathways participate in stemness, Src activity appears as a key contributor [49]. Indeed, the cancer stem cell marker, CD133, can interact and activate Src, which through the phosphorylation of FAK contributes to cancer stem cell migration [50]. At least two signalling pathways responsible for maintaining the stemness are related to Src activity in non-small cell lung cancer cells. Tescalin mediates the mutual activation of Src and IGF1R, which results in Stat3 activation and stemness [51], while EGFR/Src/Akt signalling modulates Sox2 expression and self-renewal [52]. In fact, the inhibition of Src activity with Dasatinib and PP2 reduces the clonogenic, self-renewal, and tumour-initiating capacity of pancreatic cancer stem cells [53]. Similarly, the inhibition of Src with Dasatinib, Saracatinib, PP2 or TAT-Cx43_266-283_ promotes a reduction in the expression of the inhibitor of differentiation-1 (Id1) and Sox2, with the subsequent reversion of stemness in human glioblastoma stem cells [54]. An important Src-regulated feature of CSCs is their metabolic plasticity, which allows them to survive in the ever-changing tumour microenvironment by conveniently shifting between different metabolic pathways used in energy production and catabolism [55]. These data, together with many more studies on this field, suggest that Src participates in stemness through several mechanisms triggered by a variety of signals present in different types of tumours. Therefore, maintaining the stemness should be considered as an important outcome of the high activity of Src frequently found in cancer.

## Src regulates glucose uptake and glycolysis

The transformed metabolic phenotype found in many tumours described in the late 1920s by Getty and Carl Cori [56, 57] and Otto Warburg [58, 59] included increased glucose uptake and metabolism. Glucose metabolism can be anabolic as well as catabolic, providing fuel and precursors for many if not most cell activities. To allow for the necessarily flexible and fine-tuned regulation of glucose metabolism, the implicated enzymes exhibit many levels of regulation, such as selective tissue expression, substrate affinity and specificity, modulable enzyme kinetics, subcellular localisation, and post-transcriptional modifications. Although beautiful, this intricacy can complicate the interpretation and generalisation of experimental results.

**Fig. 3:**
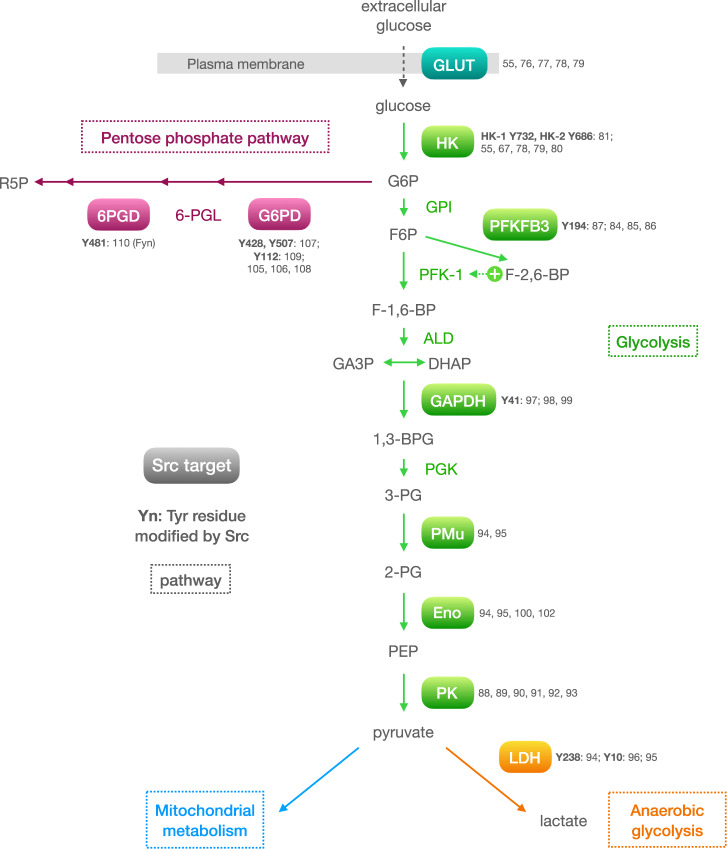
Overview of glucose metabolism proteins regulated by Src. Solid ovals represent proteins regulated by Src. Metabolic pathways are highlighted in different colours: blue, glucose transporter; green, glycolysis; orange, anaerobic glycolysis, and pink, the pentose phosphate pathway. References relating to Src regulation of each protein are indicated next to it. The tyrosine residues phosphorylated by Src are indicated together with the corresponding reference. Glycolytic metabolites: G6P glucose-6-phosphate, F6P fructose-6-phosphate, F-1,6-BP fructose-1,6-bisphosphate, F-2,6-BP fructose-2,6-bisphosphate, DHAP dihydroxyacetone phosphate, GA3P Glyceraldehyde-3-phosphate, 1,3-BPG 1,3-bisphosphoglycerate, 3-PG 3-phosphoglycerate, 2-PG 2-phosphoglycerate, PEP phosphoenolpyruvate. Pentose phosphate pathway metabolites: 6-PGL 6-phosphogluconolactone, R5P 5-ribulose-phosphate. Enzymes: HK hexokinase, GPI glucose-6-phosphate isomerase, PFK-1 phosphofructokinase 1, PFKFB3 6-phosphofructo-2-kinase, GAPDH glyceraldehyde 3-phosphate dehydrogenase, PGK phosphoglycerate kinase, PMu phosphoglycerate mutase, ENO enolase, PK pyruvate kinase, LDH lactate dehydrogenase, G6PD glucose-6-phosphate dehydrogenase, 6PGD 6-phosphogluconate dehydrogenase.

Sound evidence of Src-mediated modification of glycolysis arose very early in the history of Src research. A series of seminal works in the 1970s showed that cell transformation by Rous sarcoma virus (i.e., Src activity) induced a marked increase in glycolytic activity [60–62], mimicking the transformed metabolic phenotype found in many tumours. Since then, Src has been found to modulate glycolysis via different mechanisms, including regulation of master glycolytic transcription factors (*HIF-1ɑ* [63, 64] and *MYC* [65]), insulin secretion [66, 67], modulation of glycolytic enzyme activity by phosphorylation (HK, PFKFB3, G6PD), and through well-known Src substrates lying at the heart of energy metabolism and cancer, such as the PI3K-AKT-mTOR axis [68–70] and EGFR [71]. This section discusses many instances of mostly direct regulation of glycolysis by Src, one glycolytic protein at a time (see Fig. 3 for an overview).

### Glucose transporter

Glucose is imported into the cell by glucose transporters (GLUTs), of which there are 14 isoforms in humans [72]. Two isoforms are the most relevant and frequently overexpressed in cancer [73]. GLUT-1 (*SLC2A1*), the most ubiquitous and abundant isoform [74], and GLUT-3 (*SLC2A3*), the ‘neuronal’ isoform, which shows the highest affinity for glucose [75]. In the 1980s, early work linked higher glycolysis rates induced by *v-Src* transformation (the constitutively active form of Src) to increased levels of GLUT-1 mRNA and protein, although the underlying mechanism(s) remained unknown [76, 77]. Since then, Src activity has been shown to regulate GLUT expression through two transcription factors: in astrocytes (nervous system cells) and GBM cells (their ‘tumoral counterpart’), Src activity can modulate the levels of GLUT-1 and GLUT-3 through HIF-1α protein levels [55, 78]; in breast cancer cells, Src inhibition can decrease *MYC* expression resulting in decreased GLUT-1 mRNA and protein levels [79].

### Hexokinase

Intracellular glucose is phosphorylated by hexokinases (HKs) to glucose 6-phosphate (G6P, which is trapped into the cell), the first rate-limiting step of glycolysis (Fig. 3). Functionally, Src activity is known to regulate the expression and activity of hexokinases, most notably the major 1 and 2 isoforms (HK-1 and HK-2), in healthy as well as cancerous cells [55, 78–80], yet it was not until recently that a direct activation of HK-1 and HK-2 by Src phosphorylation was reported [81]. Phosphorylation of HK-1 and HK-2 by Src on residues Y732 and Y686, respectively, enhanced glycolysis and efficiently stimulated their catalytic activities, in the case of HK-1 by enhanced enzyme kinetics (including initial velocity [*V*_*O*_], maximum velocity [*V*_*m*_] and substrate affinity [*K*_*m*_]) and changes in oligomerization status. In the context of cancer, the authors showed that Src-stimulated tumorigenesis and metastasis was dependent on HK activity using different approaches, such as mutations in Src phosphorylation site of either HK1 or HK2 and hexokinase silencing in xenograft mouse models [81]. In agreement with these results, decreased levels of GLUT-3, HK-2 and tumorigenicity in GBM mouse models have been found upon Src inhibition [55, 82]. Finally, hexokinase IV (better known as glucokinase) has also been proven to change its activity and subcellular localisation in response to Src downregulation in insulin-producing cells [67].

### Phosphofructokinase

The resulting product of glucose phosphorylation, G6P, is isomerised to yield fructose-6-phosphate (F6P), but the specific rate-limiting step in the glycolytic pathway is the irreversible phosphorylation of F6P to fructose-1,6-bisphosphate (F-1,6-BP) by phosphofructokinase-1 (PFK-1). PFK-1 is allosterically activated by a different derivative of F6P, namely fructose-2,6-bisphosphate (F-2,6-BP), produced by 6-phosphofructo-2-kinase/fructose 2,6-bisphosphatase (PFK-2 or PFKFB). Among the four currently identified PFKFB isoforms (termed PFKFB1 to 4), PFKFB3 is the strongest glycolysis-inducer and is upregulated in many human cancers [83].

Intriguingly, PFKFB activity was found to be modulated by Src more than 35 years ago [84]. Although the authors noted the possibility that PFKFB phosphorylation by a tyrosine kinase (e.g., Src) could be the underlying mechanism, they eventually proposed an indirect mechanism involving PKC and transcriptional activity [85]. It has been reported thereafter that the absence of bisphosphatase activity strongly suggests that PFKFB3 was the predominant isoform in these samples [86]. Recently, the same group that established the HK regulation by Src identified PFKFB3 as a potential Src interaction candidate by mass-spectrometry screening [87]. They subsequently found that the N-terminal domain of PFKFB3 interacts with the SH1 domain of Src, resulting in Src phosphorylation of PFKFB3 at Tyr194. This modification induced increased cell glucose uptake, glycolysis and pentose phosphate pathway activity compared to baseline (inactive Src) and to PFKFB3-Tyr194Phe phospho-deficient cells. As expected, the PFKFB3-Tyr194Phe mutation impaired proliferation, migration, and xenograft formation. In vivo, PFKFB3-Tyr194Phe knock-in mice exhibited decreased glycolysis and attenuated spontaneous tumorigenicity. Furthermore, the levels of active (i.e., phosphorylated) PFKFB3 and Src were found to correlate in clinical tumour samples [87].

### Pyruvate kinase

Pyruvate kinase (PK), contrary to what its name suggests, catalyses the conversion of phosphoenolpyruvate (PEP) into pyruvate. As with other glycolytic enzymes, the PK isoenzyme PKM2 (the ‘muscle’ isoform, also expressed in many other cell types) was promptly identified as a Src substrate in the 1980s, but, in contrast to most other glycolytic enzymes, PKM2 phosphorylation by v-Src led to decreased affinity for PEP and more rapid ATP-mediated inactivation; in other words, Src phosphorylation inhibited PKM2 activity [88, 89]. Indeed, PKM2 is found mainly as a tetramer in healthy cells, which is the active PKM2 conformation and funnels glucose to pyruvate for energy production via mitochondrial oxidation. However, in cancer cells most PKM2 is found as a dimer—the so-called ‘tumour’ PKM2—due to (among other mechanisms) [90] phosphorylation mediated by oncoprotein kinases such as Src [88, 89, 91]. The tumour PKM2 dimer exhibits inhibited PKM2 enzyme activity, shunting glycolytic intermediaries towards anabolic processes to support rapid cell proliferation by a process that can be induced by Src phosphorylation of PKM2 [92, 93].

### Lactate dehydrogenase

Lactate dehydrogenase (LDH) catalyses both the reduction of pyruvate into lactate (LDH isoform A, LDHA), and the oxidation of lactate into pyruvate (LDH isoform B, LDHB). LDH, as well as enolase (discussed below) and phosphoglycerate mutase (PMu), was identified as a v-Src substrate in the early 1980s [94, 95]. These authors established that LDH was phosphorylated at Tyr238 by Src (RSV infection) in chick embryo cells (a common system at the time), although its functional impact remains to be explored. In addition, a 2017 report found that Src – as well as HER2 – phosphorylated LDHA at Tyr10 inducing a more active tetramer conformation that provided anti-anoikis (a form of anchorage-dependent cell death) and pro-invasive and metastatic advantages to breast cancer cells [96].

### Other glycolytic enzymes

Two other glycolytic enzymes have been found to be Src substrates, namely glyceraldehyde 3-phosphate dehydrogenase (GAPDH) and enolase. Although Src can phosphorylate GAPDH at Tyr41 [97], the outcome of this modification has been linked to vesicle trafficking [98] and the DNA damage response [99], not (yet) to glycolysis. As for enolase, in the early 1980s two different groups reported enolase phosphorylation by v-Src [94, 95, 100] and the mild enzyme kinetic effects ensuing this modification [100]. In spite of the prominent role of enolase in glycolysis and in several other cellular processes (such as tissue remodelling [101]), most of the literature linking Src and enolase refers to the use of the latter as a substrate in Src kinase activity assays [102]. Given the relevance of both GAPDH [103] and enolase activity in cancer [104], we are left to wait and wonder about whether and how Src phosphorylation might influence their role in cancer biology, as it has been clearly established for other glycolytic enzymes.

## Src and Fyn modulate the pentose phosphate pathway

An alternative fate for G6P is the pentose phosphate pathway (PPP). This route metabolises G6P in two stages: first, an oxidative phase which produces nicotinamide adenine dinucleotide phosphate (NADPH), and second, a non-oxidative phase that yields carbohydrates. Therefore, the PPP produces NADPH, an intracellular antioxidant necessary for reductive biosynthesis, ribose-5-phosphate, for nucleic acid synthesis, and erythrose 4-phosphate, for aromatic amino acid synthesis.

Several studies have shown a link between Src or Fyn activity and the regulation of PPP in contexts other than cancer [105–108]. In endothelial cells, an initial report proposed that G6PD phosphorylation by Src at residues Tyr428 and Tyr507 might modulate G6PD activity [107]. In cancer cells, the group of Li et al., who discovered the direct regulation of HK and PFKFB3 by Src, found that Src phosphorylation of G6PD at Tyr112 induces kinetic changes that increase G6PD catalytic activity and PPP flux [109]. As a result, mice injected with mutant Tyr112Phe-G6PD colorectal cancer cells developed significantly smaller tumours than their G6PD wild-type counterpart. Importantly, in clinical colorectal samples, Src and G6PD abundance and activity (probed by phosphorylation levels) were found to correlate [109]. In glioma cells, upon EGFR activation, Fyn is activated causing the phosphorylation of G6PD at Tyr481, leading to enhanced PPP activity, tumour growth and radiation resistance. Indeed, in human glioblastoma patients, the phosphorylation of 6-phosphogluconate dehydrogenase, another PPP enzyme frequently upregulated in cancer cells, at Tyr481 by Fyn is associated with increased Fyn expression and with reduced survival and worse prognosis [110]. Overall, these studies indicate that at least Src and Fyn can phosphorylate PPP enzymes at different tyrosine residues subsequently increasing PPP activity (Fig. 3), which fuels DNA replication and cell death resistance contributing to tumour malignancy.

## Src within the mitochondria

**Fig. 4:**
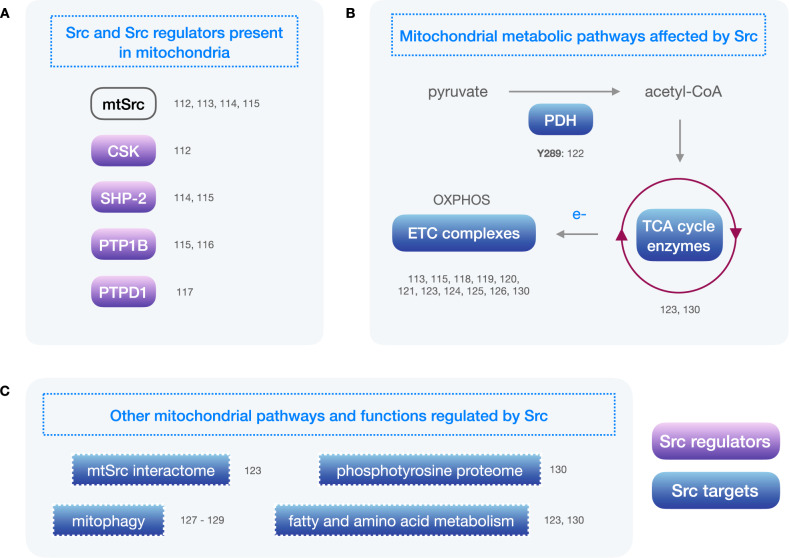
Overview of mitochondria-related proteins and pathways regulated by Src. **A** Mitochondrial Src (mtSrc) and Src-regulators present in mitochondria are shown in purple solid ovals. **B** Main mitochondrial metabolic pathways that can be regulated by Src. Blue solid ovals represent Src targets. **C** Other mitochondrial processes and pathways regulated by Src. References relating to Src regulation of each protein or pathway are indicated next to it. The tyrosine residues phosphorylated by Src are indicated together with the corresponding reference. PDH: pyruvate dehydrogenase; TCA: tricarboxylic acid; ETC: electron transport chain, OXPHOS: oxidative phosphorylation.

In the early 2000s, accruing evidence prompted the notion that mitochondrial function was regulated by protein phosphorylation, as it was being found for other organelles. Soon, attention was directed towards tyrosine phosphorylation specifically, and Abl and Src were the first tyrosine kinases demonstrated to exhibit mitochondrial localisation and activity [111–113], despite lacking canonical mitochondrial targeting sequences. Physiological regulators of Src were also promptly detected in mitochondria, including CSK [112], SHP-2 [114, 115] and PTP1B [115, 116], and even a new activator of Src was discovered, PTPD1 [117] (Fig. 4). Reported Src substrates in the mitochondria include, most notably, several complexes of the electron transport chain (ETC) [118], such as complexes I, III, IV and V [113, 115, 119–121]. Importantly, pyruvate dehydrogenase (PDH) was also described as a Src substrate [122] (Fig. 4). PDH is part of the pyruvate dehydrogenase complex, which converts pyruvate to acetyl-CoA in the mitochondria and therefore is the first step in the mitochondrial metabolism of glucose. PDH activity can be regulated through serine phosphorylation by pyruvate dehydrogenase kinases and, as reported, through tyrosine phosphorylation by Src [122]. More recently, a proximity-dependent biotin-tagging system was used to study the endogenous interactome of mitochondrial Src (mtSrc) [123]. This has led to the identification of over 50 mtSrc-interacting candidate proteins involved in different aspects of mitochondrial biology.

Initially, studies showed that an increase in mtSrc activity can lead to an increase in complex I, III and IV activity along with a decrease in complex V activity [115], and, accordingly, that a reduction in mtSrc activity can lead to a reduction in mitochondrial respiration through a decrease in complex I activity [119, 120]. Interestingly, later reports showed opposite effects of Src and mtSrc activity in mitochondrial respiration. When Src was overexpressed – in a cell-wide manner – in mouse embryonic fibroblasts (MEFs), increased phosphorylated Src levels and decreased levels of ETC complexes I and IV were found compared to wild-type MEFs, with unchanged cell proliferation [124]. Conversely, when Src was silenced in wild-type MEFs, or when Src activity was inhibited in Src overexpressing MEFs with PP2 or siRNA, the levels of ETC complexes I and IV increased compared to control conditions. Moreover, MEFs harbouring null mutations in both alleles of Src, Yes and Fyn showed decreased cell proliferation, increased ETC complex I and IV levels and increased ETC complex I/III and IV activity [124]. In the same report, metastatic liver cancer samples showed higher Src phosphorylation and decreased ETC complex levels (except for complex V) when compared to healthy liver samples, and Src inhibition with PP2 or siRNA in a liver cancer cell line led to increased ETC complex I and IV levels [124].

In triple negative breast cancer (TNBC), Park et al., using established cell lines as well as patient-derived xenografts, showed that mitochondrial fatty acid oxidase (FAO) activates mtSrc, which stimulates ETC complex activity, thereby providing ATP to maintain the Src activating phosphorylation [125]. However, this pro-tumoral mtSrc activity might be restricted to a ‘Goldilocks’ zone, as Djeungoue-Petga et al. reported that overexpression of mtSrc led to decreased mitochondrial membrane potential and respiration and cell death in several TNBC cell lines [126].

As previously mentioned, Src can regulate PDH activity, the initial step in the mitochondrial metabolism of glucose. Indeed, Src directly phosphorylates PDH on Tyr289 causing decreased PDH activity and ROS production in vitro and decreasing metastatic burden in vivo [122]. Importantly, these authors showed that combinatorial therapy consisting of Src inhibition and pro-oxidative agents had a synergistic anti-proliferative effect on breast cancer cells [122].

Mitophagy, or mitochondrial autophagy, is a process by which defective or unwanted mitochondria are selectively targeted for autophagy and degraded [127]. Several proteins, many of them susceptible to regulation by phosphorylation, finely regulate mitophagy in response to environmental as well as cell-intrinsic cues, including FUNDC1, which does so in response to cell stress such as hypoxia or mitochondrial depolarisation [127]. Under unstressed physiological conditions, FUNDC1-mediated mitophagy is inhibited by phosphorylation at Tyr18 by Src. Under hypoxia, FUNDC1 is dephosphorylated resulting in mitophagy induction. However, when Src is present, FUNDC1 phosphorylation is preserved, inhibiting FUNDC1-mediated mitophagy in response to hypoxia [128]. Nonetheless, Src does not work alone in this duty. FUNDC1 has two phosphorylation sites, Ser13 (phosphorylated by casein kinase-2) and Tyr18 (phosphorylated by Src), that functionally cooperate to regulate mitophagy. Indeed, inactivation of either Src or casein kinase-2 alone is not sufficient to activate mitophagy, while inhibition of both kinases strongly activates FUNDC1-mediated mitophagy [129].

In brief, several reports positively correlate Src activity with mitochondrial metabolism [115, 119, 120, 125] while others show the opposite trend [122, 124, 126]. Interestingly, in all cases Src activity is related with a pro-tumoral role. Several processes could underlie these a priori contradictory reports. Beyond experimental differences, such as different subcellular localisation of Src overexpression, physiological explanations across cell types might include differences in metabolic plasticity – the ability to switch metabolic pathways in response to intrinsic or extrinsic changes to maximise survival and proliferation – or differential expression of other SFKs leading to compensatory activity upon Src inhibition. Importantly, the presence and activity of different Src interacting partners, a factor often overlooked and/or understudied, might contribute greatly to the outcome of Src inhibition.

In the 2020 s, the group of E. Hébert-Chatelain has added extensive and crucial information to understand the role of Src in mitochondrial and cell metabolism using omics technologies [123, 130]. They examined the mitochondrial phosphoproteome and metabolome in Src + /+ and Src−/− mice either fed ad libitum or after fasting for 24 h. Src deletion led to impaired ETC activity and mitochondrial metabolism in several organs, along with accumulation of glucose and mitochondrial metabolites. These authors also found changes in the mitochondrial phosphoproteome and heterogenous ETC activity, oxygen consumption and metabolite abundance depending on Src phenotype and feeding state [130]. Another important contribution from this group, that supports further research in this field, is the identification of 51 candidate proteins of the mtSrc interactome involved in the tricarboxylic acid cycle, OXPHOS, cristae biology, fatty acid and amino acid metabolism and mitochondrial organisation and transport [123], highlighting the role of Src activity in the regulation of mitochondrial metabolism.

## Therapeutic opportunities of Src inhibition

Because of the prominent role of Src in cancer, several Src inhibitors have been studied in preclinical models and some of them have successfully reached clinical use. Among them, ATP-competitive Src inhibitors – Dasatinib, Saracatinib or Bosutinib – are the most extensively studied in preclinical models and clinical trials and the use of some of them, such as Dasatinib, has been approved for hematologic tumours (recently reviewed in [131]). These inhibitors bind to the ATP-binding site in Src, which is highly conserved among tyrosine kinases. The lack of kinase specificity has been exploited to target simultaneously several oncogenic kinases, but it has also been associated with undesired side effects. Despite the good results in preclinical models, the results from clinical trials with ATP-competitive Src inhibitors, such as Dasatinib, Saracatinib or Bosutinib, alone or in combination, have been discouraging so far [131]. Most evidence suggests that higher specificity and ability to reach tumour cells, reduction in side effects and drug resistance mechanisms as well as finding predictive response biomarkers is required for successful clinical results [131].

Interestingly, new appealing strategies in the development of Src inhibitors are emerging. For instance, targeting the peptide substrate binding site instead of the ATP binding site within the kinase domain of Src (Fig. 1). Potential advantages include higher kinase inhibition selectivity due to the unique sequence of the peptide substrate site, and greater binding efficacy since the inhibitor will not need to compete with mM intracellular concentrations of ATP [132]. Indeed, Src inhibitors KX2-391 and KX2-361 target the peptide substrate site at nM potencies and have selectivity among tyrosine kinases. These peptide substrate-competitive Src inhibitors are progressing in clinical trials for advanced malignancies refractory to conventional treatments (KX2-361; https://clinicaltrials.gov/ct2/show/NCT02326441) and for topical treatment against actinic keratosis (KX2-391; https://clinicaltrials.gov/ct2/show/NCT02838628).

Drug-resistance is another obstacle for a successful clinical outcome with Src inhibitors. In a recent study an interesting drug-resistance mechanism developed by ATP-competitive inhibitors has been revealed [133]. The binding of ATP-competitive inhibitors to Src allosterically disassembles Src to the open state with higher propensity to form a complex with Src substrates. If inhibitor concentration is reduced or if cells acquire a drug-resistant mutation, Src substrates will be readily phosphorylated, activating the Src signalling pathway. Consequently, the activation of Src and its downstream phosphorylation cascade can be paradoxically induced by ATP-competitive inhibitors. To prevent the relief of Src autoinhibition promoted by classical Src inhibitors, Temps et al. have developed the Src inhibitor, eCF506, which binds and locks the closed inactive conformation of Src [134]. This mechanism has the advantage of inhibiting the kinase activity as well as the protein binding to SH3 and SH2 domains in Src. Indeed, eCF506 decreases Src activity and the phosphorylation of the Src-binding protein FAK, which contrasts with some studies reporting that ATP-competitive Src inhibitors may facilitate FAK binding to Src and the subsequent phosphorylation [133, 134].

Another approach employed to design Src inhibitors is the recapitulation of the cellular mechanism for Src inhibition. As described in Fig. 1, some proteins such as CBP or Cx43 recruit Src together with its endogenous inhibitors, which causes Src inhibition. The Cx43 mimetic peptide, TAT-Cx43_266-283_, acts as a docking platform for Src, CSK and PTEN and consequently inhibits Src activity [27]. TAT-Cx43_266-283_ inhibits the oncogenic activity of Src and exerts important anti-tumoral effects in several preclinical models of glioblastoma in vitro, ex vivo, and in vivo, including freshly removed surgical specimens from patients [54, 135]. Tumour cell proliferation, survival, migration, invasion, metabolic plasticity and autophagy are impaired by TAT-Cx43_266-283_ enhancing the survival of GBM-bearing mice [55, 82, 136]. One of the main advantages of the Src inhibitor TAT-Cx43_266-283_ is that its effects are specific for the glioma stem cell subpopulation, with no effects on healthy brain cells. The cell specificity may depend on the levels of Src activity in each cell type, since TAT-Cx43_266-283_ recruits the open and active conformation of Src. Indeed, the toxicity of TAT-Cx43_266-283_ for neurons and astrocytes is much lower than that exerted by the ATP-competitive Src inhibitor Dasatinib [82]. In addition, because TAT-Cx43_266-283_ promotes the transition from the open to the closed Src conformation, both the activity and the scaffolding properties of Src are impaired, as judged by the reduction in Src activity and FAK phosphorylation promoted by TAT-Cx43_266-283_ [135].

One important conclusion to be drawn from the studies performed with Src inhibitors is that the inhibition of Src, even when using the same inhibitor and resulting in a reduction in Src activity, can impact different signalling pathways in different cell types [137]. Because Src-mediated pathways can act both in co-operation or crosstalk with other signalling pathways, these results suggest that ultimately, the effects of Src inhibition will depend on the level and activity of the repertoire of Src partners present in each cell type. Therefore, a profound study of the effect of each Src inhibitor on the main Src related signalling pathways – FAK, Akt, EGFR, Erk, Stat, etc – and cellular processes – proliferation, survival, migration, invasion, stemness and metabolism – should be carried out in the specific tumour model. We would like to highlight the relevance of studying the effects of Src inhibitors on metabolism because of the relevance of metabolic plasticity for drug resistance [138] as well as the possible side-effects due to metabolic alterations in non-cancer cells [130]. In addition, an intense metabolic cooperation has been described between tumoral cells and their microenvironment: disrupting this metabolic crosstalk might be critical to impair tumour growth. The study of the effect of Src inhibitors on different metabolic pathways in tumour cells as well as cells from the microenvironment would enhance the possibilities to achieve clinical benefits from Src inhibitors in solid tumours.

## Concluding remarks and future perspectives

Numerous studies carried out over the last years have demonstrated that key proteins and enzymes involved in glucose uptake, glycolysis, PPP and mitochondrial metabolic pathways are Src substrates (Figs. 3, 4). Consequently, the activity of Src can modulate specific metabolic pathways required to fuel diverse cancer cell activities. Not surprisingly, the inhibition of Src activity affects cancer cell metabolism with a concomitant reduction in tumour progression [55]. As a relevant proof of the link between the effects of Src on metabolism and its oncogenic activity, mutations of key metabolic enzymes, such as HK1, HK2, PFKFB3 or G6PDH at specific Src-phosphorylation sites reduce Src-evoked tumour progression [81, 87, 109]. Moreover, emerging reports are showing that Src has roles in the metabolism of several other biomolecules beyond glucose, including lipids [139, 140] and amino acids [141, 142]. Together, these data clearly indicate that the effects of Src on cell metabolism contribute to its oncogenic effect and therefore, an integrated perspective on the role of Src on these cellular functions should be considered.

Cancer progression implies heterogenous metabolic requirements to sustain diverse cellular processes, including stemness, proliferation, migration or differentiation. As described in previous sections, Src activity is not associated to an increase or decrease in a specific metabolic pathway. Overall, Src activity can regulate diverse metabolic pathways and hence we propose that this oncoprotein is in a good position to coordinate metabolism with each specific tumour cell process. For instance, Src activity can regulate mitochondrial metabolism in cancer stem cells [55], which are highly dependent on OXPHOS [143], while it activates glucose uptake and glycolysis in proliferative cells (see ‘Src regulates glucose uptake and glycolysis’), highlighting the contribution of Src activity to the metabolic plasticity required by cancer cells during tumour progression.

The increased Src enzymatic activity found in many tumours (Fig. 2) indicates that Src should be further studied as a drug development target, despite the lack of Src mutations or gene amplification found in cancer. As mentioned in the previous section, an intense study of the effects of Src inhibitors in cancer cell metabolism as well as in the metabolism of cells from the tumour microenvironment will help to elucidate the potential benefits, side effects or resistance mechanisms caused by Src inhibitors in a clinical setting. Given the demonstrated accuracy of artificial intelligence for protein structure prediction [144] (Fig. 1), a speed up in the development of specific Src inhibitors is expected once this computational tool reaches the same benchmark for the prediction of interaction affinities and the structure of small peptides, disordered regions and specific amino acid mutations.

Although the effects of Src on metabolism were discovered many years ago, several questions remain to be answered: How do different signals that converge in Src activation result in changes in distinct cellular and metabolic processes? Do different Src-binding partners affect Src structure differentially to accommodate a specific substrate? How is Src modulation of metabolism integrated with that of other metabolic sensors and regulators, such as AMPK or mTOR? This review is focused on Src, however, the effect of other SFK members (such as Yes, Fyn or Fgr) on metabolism has been reported, as we have discussed. Indeed, several interesting questions are still unanswered: What is the contribution to metabolism of each SFK member (e.g., is there functional redundancy, as sometimes found for other SFK functions)? Is it important to design specific inhibitors for some SFK members? The answers to these and other questions will give a more complete view of Src and SFK biology, which is required to develop new and more successful therapies that target Src in cancer.

In brief, the studies summarised in this review—and many others that we were unable to cite due to space limitations—indicate that Src can orchestrate glucose metabolism to fuel a great variety of signalling pathways and cellular processes, including the adaptation of cellular metabolism required for each cell activity during tumour progression. Hence, similarly to other well-known signalling molecules, including AKT, AMPK, mTOR or HIF-1α, Src might be regarded as a master regulator of glucose metabolism and coordinator of metabolism with different cancer cell processes.
